# Editorial: Novel Strategies for Anti-Tumor Vaccines

**DOI:** 10.3389/fimmu.2019.03117

**Published:** 2020-01-17

**Authors:** Roberto S. Accolla, Luigi Buonaguro, Cornelis Melief, Hans-George Rammensee, Michal Bassani-Sternberg

**Affiliations:** ^1^Department of Medicine and Surgery, School of Medicine, University of Insubria, Varese, Italy; ^2^Cancer Immunoregulation Unit, Istituto Nazionale Tumori, IRCCS, “Fond G. Pascale”, Naples, Italy; ^3^Department of Immunohematology and Blood Transfusion, Leiden University Medical Center, Leiden, Netherlands; ^4^ISA Pharmaceuticals, Leiden, Netherlands; ^5^Department of Immunology, Institute for Cell Biology, University of Tübingen, Tübingen, Germany; ^6^Department of Oncology, Ludwig Institute for Cancer Research, University of Lausanne, Lausanne, Switzerland

**Keywords:** tumor vaccine, HLA peptidome, tumor antigens, neoantigens, APC, T helper cells (Th)

The old dream of tumor immunologists is the construction of efficient anti-tumor vaccines to fight cancer. Anti-tumor vaccine strategies are necessarily focused, however, on patients who are already affected by the pathology in which the tumor not only has eluded the host initial immune response but often further creates suppressive mechanisms that keep counteracting the action of the immune system.

This Research Topic was intended to focus on several aspects of anti-tumor vaccinology and particularly on ways to increase the potency of anti-tumor vaccines by acting both on facilitating tumor antigen selection and presentation to cells of adaptive immunity and on reducing the effect of suppressive mechanisms on these immune responses. We are convinced, however, that to fight cancer single immune-based approaches cannot stand alone and thus vaccine approaches need to be complemented by other immune approaches.

Identification of the optimal repertoire of tumor antigens, in particular neoantigens, for the best use in anti-tumor vaccination is extensively discussed by Garcia-Garijo et al., who provide an overview of the existing strategies to identify neoantigens and to evaluate their immunogenicity. Indeed, only a small fraction of all tumor somatic non-synonymous mutations (NSM) identified represent *bona fide* immunogenic neoantigens, and even fewer mediate tumor rejection. Thus, the impact of neoantigens for vaccine purposes may be overestimated (1). A rich source of tumor antigens that are non-mutated but still highly tumor specific comes from analysis of the HLA ligandome landscape of tumors. To complement these studies, Fennemann et al. describe how personalized tumor vaccines containing multiple neoantigens can broaden and enhance the anti-tumor immune response. They also focus on the issue of the intratumor mutational landscape containing different tumor cell subclones, temporal and spatial diversity of neoantigen presentation and burden, and the relation to tumor immunogenicity, all parameters to be taken into account to improve clinical efficacy of personalized tumor vaccines.

The use of embryonic and pluripotent stem cells for the identification of additional suitable antigens to induce an optimal anti-tumor response are described by Ouyang et al. Approaches that combine an autologous induced pluripotent stem cells (iPSC) vaccine with an immune adjuvant have demonstrated great promise to elicit potent anti-tumor responses for cancer treatment. Insights on future directions are described by the authors.

Targeting tumor-associated antigens with specific antibodies in association with stimulating cytokines in novel formulations can be beneficial particularly in case of neoplastic diseases characterized by the pronounced expression of these antigens as it is the case of the unique tumor-associated form of MUC1 in pancreatic ductal carcinoma (PDA) described by Dreau et al.. Interestingly, the treatment results in infiltration of cells that can mediate ADCC function of phagocytes and reduction of suppressive regulatory cells, stressing the fact that effective anti-tumor treatment can also include a drastic modification of the tumor microenvironment.

Mimicking tumor antigens by approaches of anti-idiotype responses is revisited by Kohler et al.. They discuss advantages and limitations of this approach and explain how this old/novel strategy can be adapted in Biotech-standard production of therapeutic antibodies. As all nominal antigens, tumor antigens become immunogenic only if appropriately processed and presented by antigen presenting cells (APC). A novel therapeutic strategy of combining personalized vaccines in combination with standard therapy and anti-PD1 checkpoint inhibitors is proposed by Bassani-Sternberg et al. as a Phase1b study in resected pancreatic adenocarcinoma (PDAC) patients. The vaccine platform is based on autologous dendritic cells (DCs) loaded with mutated neoantigens and tumor-specific antigens identified through their original proteo-genomics antigen discovery pipeline. The addition of nivolumab to boost and maintain the vaccine effect underscores once more the belief that multiple immunological approaches should be used for optimal triggering and maintenance of the anti-tumor immune response.

Ameliorating and/or selecting the optimal DC subpopulation to present tumor antigens are discussed also by Zeng et al. who focus on a new type of DC, designated CD137 ligand-induced DC (CD137L-DCs), that induce strong cytotoxic T cell responses. They show that superior potency of CD137L-DCs in APC activity compared to other types of DCs is due to their intrinsic increased Akt-driven glycolysis, thus suggesting that Akt-driven glycolysis could be a therapeutic target to manipulate the function of CD137L-DCs for better clinical efficacy.

Increasing tumor antigen availability and T cell priming are crucial parameters for the efficient response to anti-cancer vaccines. Accolla et al. reviewed their work on tumor cells genetically modified by transfection with the MHC class II transactivator CIITA. These modified tumor cells can not only process and present antigens to naïve Th cells but they can also prime virgin tumor specific T cells. Their experimental approach has been extended to isolate MHC-II-bound relevant tumor peptides to formulate novel multipeptide vaccines (MHC-I + MHC-II- bound) against human hepatocarcinoma, presently in clinical trial^1^.

Alternative procedures of tumor antigen presentation and T cell priming are also discussed by Schluck et al. who concentrate on artificial APC methods employing biomaterials, highly promising tools to activate T cells and evoke robust *in vitro* and *in vivo* immune responses. In this perspective, they presented an overview of molecular cues that could be used to selectively expand T cell subsets that are beneficial for strong anti-tumor immune responses.

Ways to facilitate triggering of anti-tumor immune response *in vivo* have been also analyzed by Loeffler et al. who studied the immune response of patients undergoing radio-frequency ablation (RFA) of liver metastasis from colorectal cancers. RFA induced and/or boosted T cell responses specific for individual tumor antigens were frequently detectable, but not sufficient for the rejection of established tumors, indicating once more that combination therapies, such as immune checkpoint inhibitors should be considered.

Oncolytic virus (OV) therapy is also becoming an interesting strategy not only to treat but also to trigger and/or increase the anti-tumor immune response for vaccination purposes as Marchini et al. summarized in their review. They also provide information about OV-mediated immune conversion of the tumor microenvironment. As a case study they focus on the rodent protoparvovirus H-1PV and its dual role as an oncolytic and immunomodulatory agent.

Construction of therapeutic vaccines against tumors must take into account reduced or loss of MHC-I expression in tumor cells as an important mechanism of immune escape. Abdelaziz et al. stress this point and present intriguing new data on unexpected block of MHC-I-restricted tumor antigen presentation without reduction of cell surface expression of MHC-I molecule by using a Human Cytomegalovirus (HCMV)-based vector including a HPV E6/E7 fusion protein in a murine glioblastoma model. The molecular mechanism of lack of MHC-I presentation is not fully clear but it seems to correlate with defects of proteasome function generated by the HCMV vector. Of course this should be taken in serious considerations when approaches of tumor peptide vaccination using HCMV as host for gene sequences of tumor peptides are used.

In conclusion, we are confident that this Research Topic will help to better delineate the past and present problems related to the efficacy of anti-tumor vaccines and, based on this background, develop new ideas and strategies to improve their construction and efficacy for tomorrow.

## Author Contributions

All authors listed have made a substantial, direct and intellectual contribution to the work, and approved it for publication.

### Conflict of Interest

CM is employed by ISA Pharmaceuticals. The remaining authors declare that the research was conducted in the absence of any commercial or financial relationships that could be construed as a potential conflict of interest.

## Footnotes

^1^http://www.hepavac.eu.
